# Effects of carvedilol on human prostate tissue contractility and stromal cell growth pointing to potential clinical implications

**DOI:** 10.1007/s43440-024-00605-5

**Published:** 2024-06-11

**Authors:** Sheng Hu, A. Elif Müderrisoglu, Anna Ciotkowska, Oluwafemi Kale, Patrick Keller, Melanie Schott, Alexander Tamalunas, Raphaela Waidelich, Christian G. Stief, Martin Hennenberg

**Affiliations:** 1 Department of Urology, LMU University Hospital, LMU Munich, Munich, Germany; 2 Urologische Klinik und Poliklinik, Marchioninistr. 15, 81377 Munich, Germany

**Keywords:** Lower urinary tract symptoms (LUTS), Benign prostatic hyperplasia (BPH), Voiding symptoms, Alpha1-blocker, Carvedilol, Prostate smooth muscle contraction

## Abstract

**Background:**

Apart from antagonizing ß-adrenoceptors, carvedilol antagonizes vascular α_1_-adrenoceptors and activates G protein-independent signaling. Even though it is a commonly used antihypertensive and α_1_-adrenoceptors are essential for the treatment of voiding symptoms in benign prostatic hyperplasia, its actions in the human prostate are still unknown. Here, we examined carvedilol effects on contractions of human prostate tissues, and on stromal cell growth.

**Methods:**

Contractions of prostate tissues from radical prostatectomy were induced by electric field stimulation (EFS) or α_1_-agonists. Growth-related functions were examined in cultured stromal cells.

**Results:**

Concentration-response curves for phenylephrine, methoxamine and noradrenaline were right shifted by carvedilol (0.1–10 µM), around half a magnitude with 100 nM, half to one magnitude with 1 µM, and two magnitudes with 10 µM. Right shifts were reflected by increased EC_50_ values for agonists, with unchanged E_max_ values. EFS-induced contractions were reduced by 21–54% with 0.01–1 µM carvedilol, and by 94% by 10 µM. Colony numbers of stromal cells were increased by 500 nM, but reduced by 1–10 µM carvedilol, while all concentrations reduced colony size. Decreases in viability were time-dependent with 0.1–0.3 µM, but complete with 10 µM. Proliferation was slightly increased by 0.1–0.5 µM, but reduced with 1–10 µM.

**Conclusions:**

Carvedilol antagonizes α_1_-adrenoceptors in the human prostate, starting with concentrations in ranges of known plasma levels. In vitro, effect sizes resemble those of α_1_-blockers used for the treatment of voiding symptoms, which requires concentrations beyond plasma levels. Bidirectional and dynamic effects on the growth of stromal cells may be attributed to "biased agonism".

**Supplementary Information:**

The online version contains supplementary material available at 10.1007/s43440-024-00605-5.

## Introduction

Alpha_1_-adrenoceptor antagonists ("α_1_-blockers") are the first-line option in medical treatment of voiding symptoms suggestive of benign prostatic hyperplasia (BPH) [1, 2]. Voiding symptoms are characterized by impairment of bladder emptying and micturition, believed to result from urethral obstruction, caused by elevated prostate smooth muscle tone and prostate enlargement in BPH [2, 3]. Improvements in voiding symptoms by α_1_-blockers have been explained by inhibition of α_1_-adrenergic prostate smooth muscle contraction [2, 3]. In fact, α_1_-blockers may reduce international prostate symptom scores (IPSS) up to 50%, and enhance maximum urinary flow rates (Q_max_) up to 40% [1, 2]. Recommended by factually all guidelines for the management of male lower urinary tract symptoms (LUTS), they represent the most commonly prescribed drug class to initiate treatment of LUTS suggestive of BPH, and are applied for rapid improvement of symptoms [1, 4]. 5α-Reductase inhibitors (5-ARI) reduce prostate growth and size, and are primarily available to delay progression, complications and surgery in BPH [1]. Thus, 5-ARI are often combined with α_1_-blockers, but are associated with discontinuation rates up to 90%, resulting in progression, hospitalization and surgery [1, 5, 6]. While low adherence to medical treatment is a general problem in chronic diseases, an imbalance of side-effects and limited efficacy counteracts the patients’ expectations, and specifically contributes to discontinuation in the treatment of BPH [5, 6]. Most frequent side effects include asthenia and (orthostatic) hypotension with α_1_-blockers, and impacts on sexual function with 5ARIs, which are additive in combined treatment [1].

LUTS and BPH belong to the most common chronic diseases in elderly men, together with cardiovascular diseases, including arterial hypertension. Ongoing increases in case numbers of LUTS and BPH, driven by the combination of their age-dependent prevalence with demographic transitions mounted to increases in global numbers of patients with BPH of 70.5% from 2000 to 2019, so that the problem will gain further relevance soon [7, 8]. Likewise, polypharmacy is a growing issue in aging populations and commonly includes simultaneous treatment of hypertension and voiding symptoms. Side effects of α_1_-blockers may be limiting in LUTS treatment and include orthostatic hypotension, asthenia, and dizziness [1, 2]. Paradoxically, simultaneous treatment of hypertension and voiding symptoms, by single drugs such as α_1_-blockers, has been rarely considered. Treatment of LUTS and BPH often requires combinations, including reduction of voiding symptoms due to BPH and of storage symptoms due to overactive bladder (OAB) at once, in addition to combinations of α_1_-blockers and 5-ARI.

Carvedilol is a non-specific β-adrenergic receptor antagonist ("β-blocker"), used for the treatment of arterial hypertension, and simultaneously acts as an α_1_-adrenergic antagonist [9]. Off-target antagonism of α_1_-adrenoceptors is known from a panel of β-adrenergic ligands but usually requires concentrations out of physiological ranges [10]. In contrast, affinities of carvedilol, reported from biochemical competition assays with recombinant receptors, were in low nanomolar ranges for β- and α_1_-adrenoceptors [11]. Consequently, antagonism of α_1_-adrenoceptors appeared possible in vivo, where plasma levels range from 115 to 315 nM with standard doses [11]. Apart from the dual antagonism of β- and α_1_-adrenoceptors, carvedilol shows a unique pharmacological profile by activating β-arrestins by β-adrenoceptors, following binding of carvedilol and referred to as "biased agonism" [12]. Initially described as adapter proteins causing desensitization of G protein-coupled receptors, by uncoupling of receptors from G proteins after binding of β-arrestins to receptors, it turned out that β-arrestins themselves may cause G protein-independent signaling. Thus, the binding of carvedilol to β-adrenoceptors interrupts G protein-dependent signaling by receptor antagonism, but may simultaneously induce β-arrestin-mediated signaling by these receptors [11, 12]. Outcomes and downstream effectors of this signaling are still unknown, in particular for smooth muscle cells, and may range from regulation of growth-related functions to cellular metabolism and other functions.

Antagonism of α_1_-adrenoceptors by carvedilol has been reported from biochemical competition assays and contraction experiments with myocardial tissues and isolated vessels, but not using human smooth muscle tissues examined with carvedilol concentrations in physiological ranges. As α_1_-adrenoceptors are a most important target for medical treatment in LUTS suggestive of BPH, and carvedilol is clinically available, we were interested in exploring its efficacy and potency for antagonism of α_1_-adrenoceptors in the human prostate, and outcomes of carvedilol stimulation on growth-related functions.

## Materials and methods

### Human prostate tissues

Human prostate tissues were obtained from radical prostatectomy for prostate cancer. Prostates from patients with previous ablative surgery for BPH were excluded. This study was carried out in accordance with the Declaration of Helsinki of the World Medical Association and has been approved by the ethics committee of the Ludwig-Maximilians University, Munich, Germany (approval number 22-0827, from 10-18-2022). Informed consent was obtained from all patients. All samples and data were collected and analyzed anonymously. Accordingly, no patient data such as age, clinical data or information about preoperative medications were collected, stored or analyzed for this study, and retrospective analyses with patients’ data are impossible. Approximately 30–60 min after organ retrieval, macroscopical inspection and sampling were carried out by a pathologist. For storage and transport, prostates and samples were placed in Custodiol^®^ solution (for cardioplegia and heart transplantation, approval number 43815.00.00) (Köhler, Bensheim, Germany). For macroscopic inspection and sampling, the prostate was opened by a single longitudinal cut from the capsule to the urethra, and both surfaces were macroscopically examined for any obvious tumor infiltration. Subsequently, tissues were prepared from the transitional, periurethral zone. Prostates with macroscopically visible tumors in the periurethral zone were excluded from sampling. This was rare (< 1% of prostates), as most prostate tumors are located in the peripheral zone [13, 14]. Experiments were initiated within 3 h following sampling.

### Materials, drugs, and nomenclature

Carvedilol was obtained from Tocris (Bristol, UK; catalogue number 2685). Stock solutions were prepared with dimethylsulfoxide (DMSO; Carl Roth, Karlsruhe, Germany; catalogue number 4720.1), and stored as aliquots at − 20 °C. Separate stock solutions (1000 fold of final concentrations) were used for each carvedilol concentration, i.e. 10 µM, 100 µM, 1 mM, and 10 mM, from which 10 µl were added per organ bath chamber to obtain final concentrations of 10 nM, 100 nM, 1 µM or 10 µM carvedilol. Thus, the amount of DMSO (1 ‰) in control and carvedilol groups was the same in each experiment. Noradrenaline (catalogue number 74480-1G), phenylephrine (catalogue number P6126-10G), and methoxamine (catalogue number M6525-1G) were obtained from Sigma-Aldrich (Munich, Germany). Stock solutions of noradrenaline, phenylephrine, and methoxamine with distilled water were freshly prepared before each experiment. U46619 is an agonist of the thromboxane A_2_ receptor and was dissolved in ethanol. Stock solutions (10 mM) were stored at − 80 °C until use. U46619 (catalogue number BML-PG023-0010) and endothelin-1 (catalogue number ALX-155-001-P001) were obtained from Enzo Life Sciences (Lörrach, Germany).

### Organ bath experiments

Prostate strips (6 × 3 × 3 mm) were mounted in organ baths (model 720 M, Danish Myotechnology, Aahus, Denmark), with four chambers in each device, containing 10 ml continuously gassed (95% O_2_ and 5% CO_2_) Krebs–Henseleit solution (37 °C, pH 7.4), with the following composition: 118 mM NaCl, 4.7 mM KCl, 2.55 mM CaCl_2_, 1.2 mM KH_2_PO_4_, 1.2 mM MgSO_4_, 25 mM NaHCO_3_, 7.5 mM glucose. Preparations were stretched to 4.9 mN and left to equilibrate for 45 min. As tensions typically spontaneously decline in the initial phase after mounting, tensions were adjusted three times during the equilibration period, until a stable resting tone of 4.9 mN was attained within 45 min. Subsequently, maximum contraction induced by 80 mM KCl was assessed in each sample, by the addition of a 2 M stock solution. As soon as a maximum contraction was obtained, chambers were rinsed three times with Krebs–Henseleit solution for a total of approximately 20 min, followed by the addition of carvedilol or an equivalent amount of DMSO to controls. Cumulative concentration–response curves for α_1_-adrenergic agonists (phenylephrine, noradrenaline, methoxamine), U46619 or endothelin-1, or frequency response curves for electric field stimulation (EFS) were constructed 30 min after the addition of carvedilol or DMSO. Application of EFS in human prostate tissues simulates action potentials, resulting in contractions by the release of endogenous neurotransmitters, including noradrenaline [15]. For EFS, tissues were placed between two opposing platinum electrodes connected to a CS4 stimulator (Danish Myotechnology, Denmark). Square pulses with durations of 1 ms were applied with a voltage of 20 V, for a train duration of 10 s. EFS-induced contractile responses were studied at frequencies of 2, 4, 8, 16, and 32 Hz, with train intervals of 30 s between stimulations.

Each independent experiment included a carvedilol (i.e. one single carvedilol concentration) and a control group, with tissues in both groups being obtained from the same organ. Thus, for one independent experiment, tissue from one prostate was split into the control and carvedilol groups within the same experiment. Only one concentration-response or frequency response curve was created with each sample. If applicable, double determinations were performed. For double determinations, two organ bath chambers were examined with carvedilol, and the two others in the same device as controls with DMSO, used as a solvent for carvedilol, and with all four tissues obtained from the same prostate. From a total of 193 experiments, double determinations in both groups were possible in 157 experiments. In the remaining experiments, the amount of sampled tissues did not allow the filling of two channels for both groups, or samples did not contract with KCl, so single determinations were performed in one group, or rarely in both groups. However, each experiment included at least one sample for both groups, resulting in paired samples. Allocations of chambers to groups were changed between experiments. Indicated numbers of experiments (n) represent the number of included patients in a given series. Thus, all single experiments within a series were performed with prostates from different patients (e.g. n = 5 corresponds to 5 patients participating in this series). If sampled tissues from one prostate allowed more than one experiment, the tissue was allocated to different series, but not to independent experiments within the same series.

KCl-induced contractions, assessed before the addition of DMSO or carvedilol, varied between samples (supplementary materials Fig. 1A), attributed to the high heterogeneity of human prostate tissues from radical prostatectomy. Across all experiments, groups and series, contractions induced by KCl mounted to 3.8 mN [3.4 to 4.1], with contractions in all control groups mounting to 3.5 mN [3 to 5], and to 4 mN [3.5 to 4.5] in all carvedilol groups (supplementary materials Fig. 1A). In all experiments with α_1_-adrenergic agonists, KCl-induced contractions in carvedilol groups were similar to corresponding control groups (supplementary materials Fig. 1B–D).

Agonist- and EFS-induced contractions are expressed as a percentage of 80 mM KCl-induced contractions. The maximum possible contractions (E_max_), concentrations inducing 50% of maximum agonist-induced contraction (EC_50_), and frequencies (f) inducing 50% of maximum EFS-induced contraction (Ef_50_) were calculated by curve fitting, separately for each single experiment using GraphPad Prism 6 (GraphPad Software Inc., San Diego, CA, USA). Sigmoidal concentration-response curves, and frequency response curves were fitted by non‐linear regression, without predefined restrictions for top, bottom or EC_50_ values, without automatic outlier elimination or weighting, by ordinary fit. Error messages, sent by the software if results from curve fitting are suspected as "ambiguous" or non-plausible occurred in four experiments, including one experiment with methoxamine and two with phenylephrine, all with 10 µM carvedilol, where concentration-response curves were still fully in exponential uphill phases at the highest agonist concentration, and one experiment with phenylephrine and 1 µM carvedilol, where the curve was still at the beginning of the uphill phase at the highest agonist concentration. Values calculated from these four experiments were labeled as ambiguous by the software and were in fact fully implausible (including six-digit E_max_ values, and positive logEC_50_ values), and consequently handled as follows. Concerning EC_50_ values were replaced by the highest applied agonist concentration (i.e. − 3 or − 4), as a broad approximation, which may become plausible from inspection of single curves, at least for experiments ending in the fully exponential phase. Consequently, E_max_ values for these experiments were estimated as the two-fold contractions seen at the highest applied agonist concentration. In scatter plots, points representing these values have been marked by gray color.

To estimate the affinity of carvedilol for α_1_-adrenoceptors in our experiments, “apparent” pA_2_ values were calculated as the sum of the negative decadic logarithm of the carvedilol concentration, and the right shift in concentration-response curves for α_1_-adrenoceptors, expressed as negative decadic logarithm: apparent pA_2_ = p[carvedilol] + (pEC_50_ α_1_-agonist controls – pEC_50_ α_1_-agonist with carvedilol). Values were calculated separately for each single experiment.

### Cell culture

Cell culture experiments were carried out in WPMY-1 cells (RRID:CVCL_3814), an SV40 large-T antigen-immortalized cell line from the stroma of a human prostate without prostate cancer, and show characteristics of prostate smooth muscle cells [16, 17]. Cells were purchased from the American Type Culture Collection (ATCC; Manassas, VA, USA; catalogue number CRL-2854), and cultured in RPMI 1640 (Gibco, Carlsbad, CA, USA; catalogue number 21875091) supplemented with 10% fetal calf serum (FCS) (Gibco; catalogue number A5256701) and 1% penicillin/streptomycin at 37 °C with 5% CO_2_. The medium was changed to an FCS-free medium 24 h prior to the addition of carvedilol or DMSO to cells.

### Colony formation assay

Carvedilol or DMSO was added 24 h after transfer of WPMY-1 cells to 6-well plates (100 cells/well), followed by culture for a further 13 days. Finally, plates were washed five times with cold water, stained for 30 min using 0.4% sulforhodamine B solution at room temperature, and washed five times with 1% acetic acid before taking photos. From these photos, all visible colonies were counted, and the results are expressed as the number of colonies (n) per well. Colony-covered areas, expressed as percentage of whole well areas were quantified using Image J (National Institutes of Health, Bethesda, Maryland, USA), from pictures of whole wells.

### Viability assay

The viability of WPMY-1 cells was assessed using the Cell Counting Kit-8 (CCK-8) (Sigma-Aldrich, Munich, Germany; catalogue number 96992), 24, 48 or 72 h after the addition of carvedilol or DMSO. Cells were seeded in 96-well plates (5000 cells/well), and cultured for 24, 48 or 72 h with carvedilol or DMSO. Finally, 10 μL of 2-(2-methoxy-4-nitrophenyl)- 3-(4-nitrophenyl)-5-(2,4-disulfophenyl)-2H-tetrazolium monosodium salt (WST-8) from the kit were added, and absorbance in each well was measured at 450 nm after incubation for 2 h at 37 °C, and values are reported as optical densities (OD).

### Proliferation assay

The proliferation rate of cells was assessed using the 5-ethynyl-2'-deoxyuridine- (EdU-)based EdU-Click 555 proliferation assay kit (Baseclick, Tutzing, Germany; catalogue number BCK-EdU555IM100), which was applied according to the manufacturer’s instructions. In this assay, the incorporation of EdU into the DNA of proliferating cells is visualized by detection with fluorescing 5-carboxytetramethylrhodamine (5-TAMRA). For each group, 10,000 cells were placed per well of 16-well chambered coverslip (Thermo Scientific, Waltham, MA, USA), and cultured in FCS-free medium for 24 h. Subsequently, DMSO or carvedilol was added, and cells were cultured for a further 12 h or 36 h, before the medium was replaced by 10 mM EdU solution in FCS-free smooth muscle cell medium containing carvedilol or DMSO, followed by culture for another 12 h before fixing and resulting in exposure to DMSO or carvedilol for 24 h or 48 h. Cells were fixed with ROTI^®^ Histofix 4% solution (Roth, Karlsruhe, Germany). Counterstaining of all nuclei was performed with DAPI. Finally, detection was performed by fluorescence microscopy (excitation: 546 nm; emission: 479 nm) using a laser scanning microscope (Leica DMI8 (TCS SP8 X), Wetzlar, Germany), with a 40 × oil objective. Images (4.46 × 4.46 mm, pixel size 4.36 µm × 4.36 µm) were taken using the Leica Application Suite X software (3.5.723225). A total of five independent experiments were performed, with each single experiment containing a control group and groups for all examined carvedilol concentrations. In each independent experiment, five pictures were taken per group, from five different, randomly selected regions, resulting in means from five-fold determinations in each single experiment, and a total of 25 images analyzed per group across all five experiments. Stained nuclei were counted using “Image J” (National Institutes of Health, Bethesda, Maryland, USA), and the proliferation rate (%) is reported as the percentage of EdU-stained nuclei from all nuclei (i.e. EdU- and DAPI-stained).

### Data and statistical analyses

Data in concentration and frequency response curves are means ± standard deviation (SD). Values from curve fitting and cell culture experiments are presented as single values from each experiment, together with means in scatter plots. Increases in EC_50_ values for agonists, expressed as mean difference (MD) as logM have been calculated from paired groups for each experiment and are reported as means with 95% confidence intervals (CIs). Decreases in E_max_ values for EFS-induced contractions are expressed as absolute values (% of KCl-induced contractions) and are reported as means with 95% CI together with contractions in controls. Decreases at single concentrations of endothelin-1 have been calculated by referring values with carvedilol to values in the corresponding control group in each experiment, and are reported as means with 95% CI. Effect sizes in cell culture experiments were calculated by referring values of carvedilol groups in each experiment to the mean of corresponding control groups, and are reported as fold of controls or as percentage decreases from controls, as means with 95% CI. Calculation of 95% CIs and statistical analyses were performed using GraphPad Prism 6. Comparison of whole concentration-response curves was performed by two-way analysis of variance (ANOVA), including multiple comparisons (Sidak’s multiple comparisons test) at each agonist concentration within curves. Comparison of whole frequency response curves was performed by mixed ANOVA, including multiple comparisons (Sidak’s multiple comparisons test) at each frequency within curves. E_max_, EC_50_ and Ef_50_ values, as well as KCl-induced contractions in carvedilol groups and corresponding controls represented paired values (as pairs of control and carvedilol values were obtained from the same tissues) were compared by a paired Student’s t-test. KCl-induced contractions across all tissues, all control groups, and all carvedilol groups were compared by one-way ANOVA with Tukey’s test. Carvedilol groups in cell culture experiments were compared to controls by one-way ANOVA with Dunnett’s test. P values < 0.05 were considered significant. P values, F values, df, t, and interactions for main effects are reported in the text [18], while all values are summarized in supplementary materials Table 1, together with interactions and with main results from posthoc tests applied to concentration and frequency response curves. The present study and analyses show an exploratory design, as typical features of a strictly hypothesis-testing study were lacking, including biometric calculation of group sizes, blinding, or a clear preset study [19]. Consequently, p values reported here need to be considered as descriptive, but not as hypothesis-testing [19]. Minimum group sizes were pre-planned as n = 5 for each series, to allow calculation of descriptive p values. Thus, the series were discontinued after five independent experiments, if it was obvious that no effect could be expected, or if p values were < 0.05 between groups. If these initial results were not conclusive, i. e. suggested a possible carvedilol effect despite p values > 0.05, the series was continued and analyzed again. Specifically, increasing group sizes after five initial experiments were applied to experiments with phenylephrine and 1 µM carvedilol (n = 6), EFS and 100 nM carvedilol (n = 6), and U46619 and 10 µM carvedilol (n = 7). This procedure was in line with the explorative character, and as it is reported here in detail [19]. Interim analyses were limited to frequency and concentration-response curves and did not include curve fitting, which was performed after the completion of the series. No data or experiments were excluded from analyses.

## Results

### Effects of carvedilol on phenylephrine-induced contractions

**Fig. 1:**
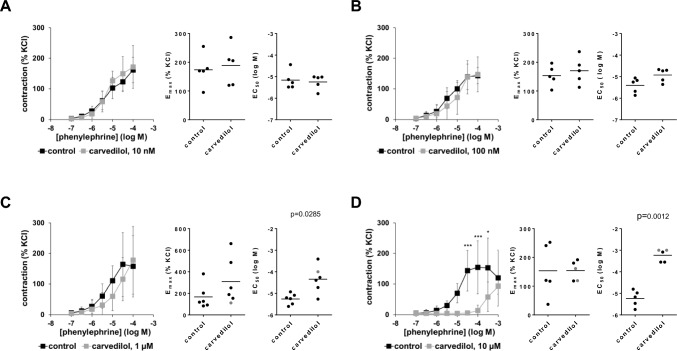
Effects of carvedilol on phenylephrine-induced contractions of human prostate tissues. Contractions were induced by phenylephrine, 30 min after the addition of 10 nM carvedilol (**A**), 100 nM carvedilol (**B**), 1 µM carvedilol (**C**) or 10 µM carvedilol (**D**), or of an equivalent amount of dimethylsulfoxide (DMSO, controls). Data from 5 independent experiments (**A**,**B**,**D**), and 6 independent experiments (**C**), where tissues from 5 (**A**,**B**,**D**) or 6 patients (**C**) were split to both groups of a panel (i.e. carvedilol and control), resulting in paired samples. Data are shown as means ± standard deviation from all experiments in concentration–response curves (two-way ANOVA), and all single E_max_ and EC_50_ values from all experiments (calculated by curve fitting) (paired Student’s *t*-tests) in scatter plots. *p < 0.05, **p < 0.01, ***p < 0.001, ****p < 0.0001 between control and carvedilol. Values based on approximation due to ambiguous values after curve fitting are marked in gray

Carvedilol caused concentration-dependent right shifts of concentration-response curves for phenylephrine, without decreasing E_max_ values calculated by curve fitting by any applied carvedilol concentration (Fig. 1A–D, supplementary materials Fig. 2A). Concentration-response curves were unaffected with 10 nM carvedilol (F_1,28_ = 0.8089, p = 0.3761 for carvedilol vs. control; interaction F_6,28_ = 0.6585, p = 0.6382; Fig. 1A), slightly right shifted around half a magnitude with 100 nM (F_1,28_ = 1.928, p = 0.1759 for carvedilol vs. control; interaction F_6,28_ = 0.6302, p = 0.7049; Fig. 1B) or less than one magnitude with 1 µM (F_1,35_ = 4.74, p = 0.0363 for carvedilol vs. control; interaction F_6,35_ = 1.849, p = 0.1179; Fig. 1C), and righshifted around two magnitudes with 10 µM carvedilol (F_1,36_ = 31.0, p < 0.0001 for carvedilol vs. control; interaction F_8,36_ = 3.489, p = 0.0044; Fig. 1D). Right shifts were reflected by corresponding increases in EC_50_ values for phenylephrine, mounting to 0.48 [-0.05 to 1.01] magnitudes with 100 nM (t_4_ = 2.53; p = 0.0651), 0.92 [0.14 to 1.69] magnitudes with 1 µM (t_5_ = 3.048; p = 0.0285), and 1.96 [1.45 to 2.48] magnitudes with 10 µM carvedilol (t_4_ = 8.271; p = 0.0012) (Fig. 1B–D).

### Effects of carvedilol on methoxamine-induced contractions

**Fig. 2:**
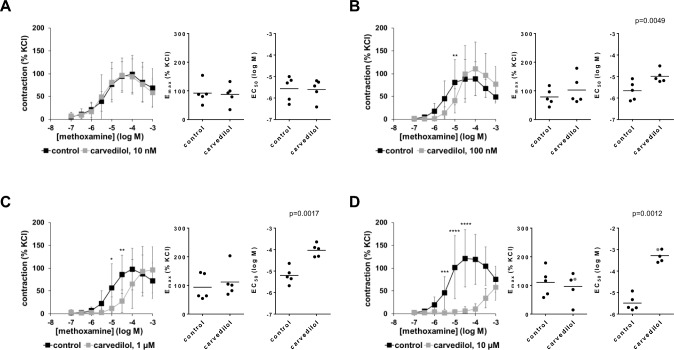
Effects of carvedilol on methoxamine-induced contractions of human prostate tissues. Contractions were induced by methoxamine, 30 min after the addition of 10 nM carvedilol (**A**), 100 nM carvedilol (**B**), 1 µM carvedilol (**C**) or 10 µM carvedilol (**D**), or of an equivalent amount of dimethylsulfoxide (DMSO, controls). Data from 5 independent experiments in each panel, where tissues from 5 patients were split to both groups of a panel (i.e. carvedilol and control), resulting in paired samples. Data are are shown as means ± standard deviation from all experiments in concentration–response curves (two-way ANOVA), and all single E_max_ and EC_50_ values from all experiments (calculated by curve fitting) (paired Student’s *t*-tests) in scatter plots. *p < 0.05, **p < 0.01, ***p < 0.001, ****p < 0.0001 between control and carvedilol. Values based on approximation due to ambiguous values after curve fitting are marked in gray

Carvedilol caused concentration-dependent right shifts of concentration-response curves for methoxamine, without affecting E_max_ values calculated by curve fitting (Fig. 2A–D, supplementary materials Fig. 2B). Concentration-response curves were unaffected with 10 nM carvedilol (F_1,36_ = 0.0521, p = 0.82 for carvedilol vs. control; interaction F_8,36_ = 0.2605, p = 0.9746; Fig. 2A), right shifted more than half a magnitude with 100 nM (F_1,36_ = 0.000253, p = 0.9874 for carvedilol vs. control; interaction F_8,36_ = 5.204, p = 0.0002; Fig. 2B), around one magnitude with 1 µM (F_1,36_ = 9.141, p = 0.0046 for carvedilol vs. control; interaction F_8,36_ = 3.368, p = 0.0056; Fig. 2C), and around 2.5 magnitudes with 10 µM carvedilol (F_1,36_ = 56,15, p < 0.0001 for carvedilol vs. control; interaction F_8,36_ = 4.902, p = 0.0004; Fig. 2D). Right shifts were reflected by corresponding increases in EC_50_ values for methoxamine, mounting to 0.67 [0.34 to 1] magnitudes with 100 nM (t_4_ = 5.627; p = 0.0049), 1.28 [0.85 to 1.7] magnitudes with 1 µM (t_4_ = 7.501; p = 0.0017), and 2.14 [1.55 to 2.73] magnitudes with 10 µM carvedilol (t_4_ = 8.267; p = 0.0012) (Fig. 2B–D).

### Effects of carvedilol on noradrenaline-induced contractions

**Fig. 3:**
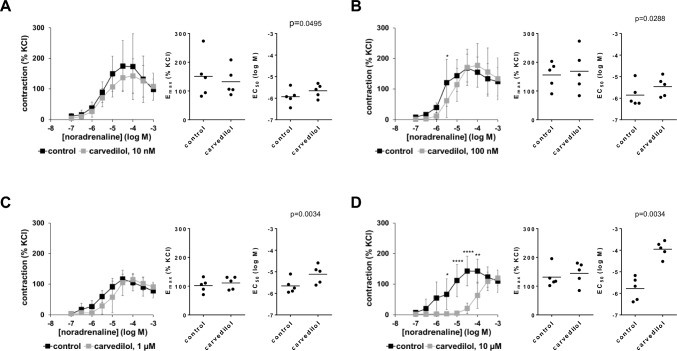
Effects of carvedilol on noradrenaline-induced contractions of human prostate tissues. Contractions were induced by noradrenaline, 30 min after the addition of 10 nM carvedilol (**A**), 100 nM carvedilol (**B**), 1 µM carvedilol (**C**) or 10 µM carvedilol (**D**), or of an equivalent amount of dimethylsulfoxide (DMSO, controls). Data from 5 independent experiments in each panel, where tissues from 5 patients were split to both groups of a panel (i.e. carvedilol and control), resulting in paired samples. Data are shown as means ± standard deviation from all experiments in concentration–response curves (two-way ANOVA), and all single E_max_ and EC_50_ values from all experiments (calculated by curve fitting) (paired Student’s *t*-tests) in scatter plots. *p < 0.05, **p < 0.01, ***p < 0.001, ****p < 0.0001 between control and carvedilol

All four examined carvedilol concentrations increased the EC_50_ values for noradrenaline, without affecting E_max_ values calculated by curve fitting, which was paralleled by concentration-dependent right shifts of concentration-response curves for noradrenaline (Fig. 3A–D). Concentration-response curves suggested slight inhibition of lacking recovery at high agonist concentrations with 10 nM carvedilol (F_1,36_ = 8.369, p = 0.0064 for carvedilol vs. control; interaction F_8,36_ = 1.010, p = 0.4461; Fig. 3A), or were right shifted around half a magnitude with 100 nM (F_1,36_ = 2.599, p = 0.1156 for carvedilol vs. control; interaction F_8,36_ = 2.539, p = 0.0265; Fig. 3B) and 1 µM carvedilol (F_1,36_ = 3.72, p = 0.0617 for carvedilol vs. control; interaction F_8,36_ = 1.769, p = 0.1159; Fig. 3C), and to nearly two magnitudes with 10 µM carvedilol (F_1,36_ = 65.56, p < 0.0001 for carvedilol vs. control; interaction F_8,36_ = 6.301, p < 0.0001; Fig. 3D). Right shifts were reflected by corresponding increases in EC_50_ values for noradrenaline, mounting to 0.28 [0 to 0.55] magnitudes with 10 nM (t_4_ = 2.787; p = 0.0495), 0.4 [0.07 to 0.73] magnitudes with 100 nM (t_4_ = 3.341; p = 0.0288), 0.75 [0.19 to 1.32] magnitudes with 1 µM (t_4_ = 6.219; p = 0.0034), and 1.82 [1.2 to 2.44] magnitudes with 10 µM carvedilol (t_4_ = 6.1; p = 0.0034) (Fig. 3B–D).

### Apparent pA_2_ values

**Fig. 4:**
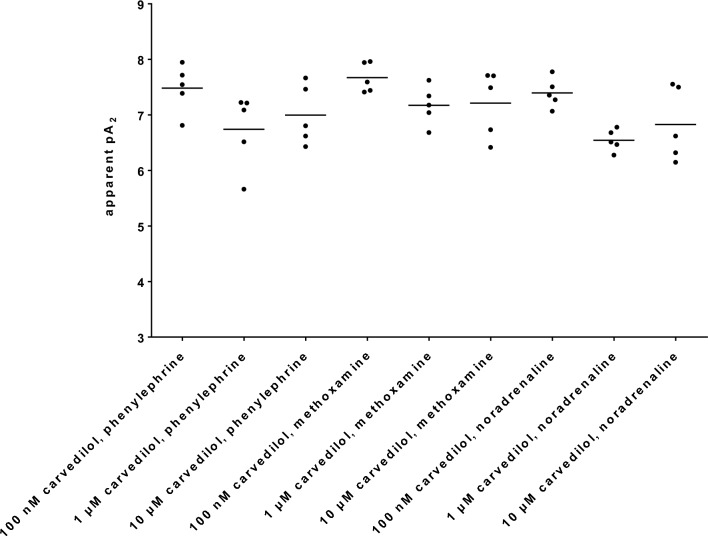
Apparent pA_2_ values of carvedilol. Apparent pA_2_ values were calculated for series with 100 nM, 1 µM and 10 µM carvedilol, applied to phenylephrine, methoxamine and noradrenaline. The calculation was based on experiments shown in Figs. 1, 2 and 3

Apparent pA_2_ values (Fig. 4) were calculated for series with 100 nM, 1 µM and 10 µM carvedilol, applied to phenylephrine, methoxamine and noradrenaline, from experiments reported above (Figs. 1, 2 and 3). Calculation was possible for all experiments in these series. Across all six series, average apparent pA_2_ values ranged between 21 and 286 nM (Fig. 4). Across series only including carvedilol concentrations of 1 µM and 10 µM, where right shifts were most obvious, average apparent pA_2_ values ranged between 63 and 286 nM (Fig. 4). Specifically, apparent pA_2_ values with 100 nM, 1 µM and 10 µM carvedilol mounted to 7.47 [6.95 to 8.01], 6.74 [5.91 to 7.57] and 6.997 [6.326 to 7.67] in experiments with phenylephrine, to 7.67 [7.34 to 8], 7.17 [6.74 to 7.61] and 7.21 [6.47 to 7.96] in experiments with methoxamine, and to 7.4 [7.07 to 7.73], 6.54 [6.3 to 6.79] and 6.83 [6.01 to 7.65] in experiments with noradrenaline (Fig. 4).

### Effects of carvedilol on EFS-induced contractions

**Fig. 5:**
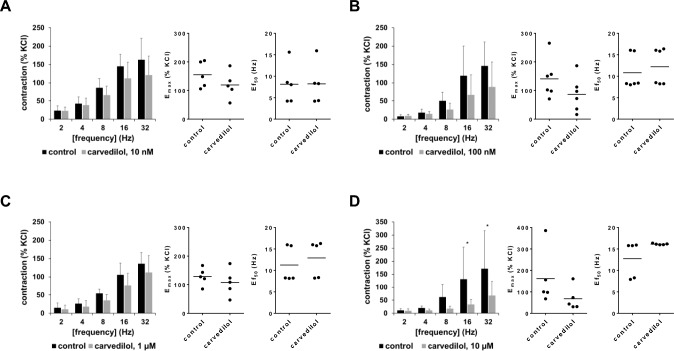
Effects of carvedilol on EFS-induced contractions of human prostate tissues. Contractions were induced by electric field stimulation (EFS), 30 min after the addition of 10 nM carvedilol (**A**), 100 nM carvedilol (**B**), 1 µM carvedilol (**C**) or 10 µM carvedilol (**D**), or of an equivalent amount of dimethylsulfoxide (DMSO, controls). Data from 5 independent experiments in (**A**,**B**,**D**), and 6 independent experiments (**B**), where tissues from 5 (**A**,**C**,**D**) or 6 patients (**B**) were split to both groups of a panel (i.e. carvedilol and control), resulting in paired samples. Data are shown as means ± standard deviation from all experiments in frequency response curves (mixed ANOVA), and all single E_max_ and Ef_50_ values from all experiments (calculated by curve fitting) in scatter plots. E_max_ and Ef_50_ values (not significant) were analyzed by paired Student’s *t*-test. *p < 0.05, **p < 0.01, ***p < 0.001, ****p < 0.0001 between control and carvedilol

All four examined carvedilol concentrations reduced EFS-induced contractions, as seen in frequency response curves, and suggested for the three highest frequencies in each series (Fig. 5A–D). A clear concentration-dependent relationship was lacking and inhibition occurred with 10 nM (mixed ANOVA for contraction: F_1,40_ = 4.427, p = 0.0417 for carvedilol vs. control; mixed ANOVA interaction F_4,40_ = 0.6728, p = 0.6148; Fig. 5A), 100 nM (mixed ANOVA for contraction: F_1,25_ = 7.293, p = 0.0122 for carvedilol vs. control; mixed ANOVA interaction F_4,25_ = 1.416, p = 0.2577; Fig. 5B) and 1 µM carvedilol (mixed ANOVA for contraction: F_1,20_ = 9.094, p = 0.0068 for carvedilol vs. control; mixed ANOVA interaction F_4,20_ = 0.7258; p = 0.5847; Fig. 5C), but inhibitions appeared largest with 10 µM carvedilol (mixed ANOVA for contraction: F1,20 = 14.15, p = 0.0012 for carvedilol vs. control; mixed ANOVA interaction F_1,20_ = 2.487; p = 0.0763; Fig. 5D). E_max_ values, calculated by curve fitting amounted to 155% of KCl-indued contractions [99 to 212] in controls for 10 nM carvedilol, 141% of KCl-indued contractions [68 to 213] in controls for 100 nM, 129% of KCl-indued contractions [91 to 166] in controls for 1 µM, and 162% of KCl-indued contractions [1 to 322] in controls for 10 µM carvedilol, which were consistently reduced in all four series. Decreases in E_max_ values (percentage points from KCl-induced contractions) amounted to − 36% [− 107 to 36] with 10 nM carvedilol (t_4_ = 1.387; p = 0.2378) (Fig. 5A), − 54% [− 136 to 28] with 100 nM (t_5_ = 1.683; p = 0.1532) (Fig. 5B), − 21% [− 81 to 39] with 1 µM (t_4_ = 0.9803; p = 0.3824) (Fig. 5C), and − 94% [− 197 to 9] with 10 µM carvedilol (t_4_ = 2.524; p = 0.0651) (Fig. 5D).

### Effects of carvedilol on non-adrenergic contractions

**Fig. 6:**
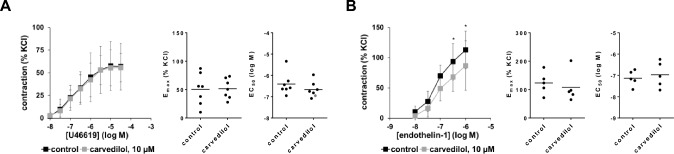
Effects of carvedilol on non-adrenergic contractions of human prostate tissues. Contractions were induced by the thromboxane A_2_ analog U46619 (**A**) or by endothelin-1 (**B**), 30 min after the addition of 10 µM carvedilol or of an equivalent amount of dimethylsulfoxide (DMSO, controls). Data from 7 independent experiments with U46619 and 5 independent experiments with endothelin-1, where tissues from 7 (**A**) or 5 patients (**B**) were split to both groups of a panel (i.e. carvedilol and control), resulting in paired samples. Data are shown as means ± SD from all experiments in concentration–response curves (two-way ANOVA), and all single E_max_ and EC_50_ values from all experiments (calculated by curve fitting). E_max_ and Ef_50_ values (not significant) were analyzed by paired Student’s *t*-test. *p < 0.05, **p < 0.01, ***p < 0.001, ****p < 0.0001 between control and carvedilol

Effects of carvedilol on concentration-response curves for U46619 and endothelin-1 were examined using a concentration of 10 µM. Concentration-response curves and E_max_ and EC_50_ values for U46619 were not changed by carvedilol (F_1,48_ = 0.497, p = 0.497 for carvedilol vs. control; interaction F_7,48_ = 0.02279, p > 0.9999; Fig. 6A). Contractions by endothelin-1 were slightly reduced by 10 µM carvedilol (F_1,20_ = 25.72, p < 0.0001 for carvedilol vs. control; interaction F_4,20_ = 1.172, p = 0.3527; Fig. 5B), amounting to decreases of 27% [− 43 to − 11] and 23 [− 53 to 7] at 0.3 µM and 1 µM endothelin-1, if contractions in carvedilol groups were referred to corresponding controls in each experiment. However, E_max_ or EC_50_ values for endothelin-1 were not changed by carvedilol (Fig. 6B).

### Effects of carvedilol on colony formation of stromal cells

**Fig. 7:**
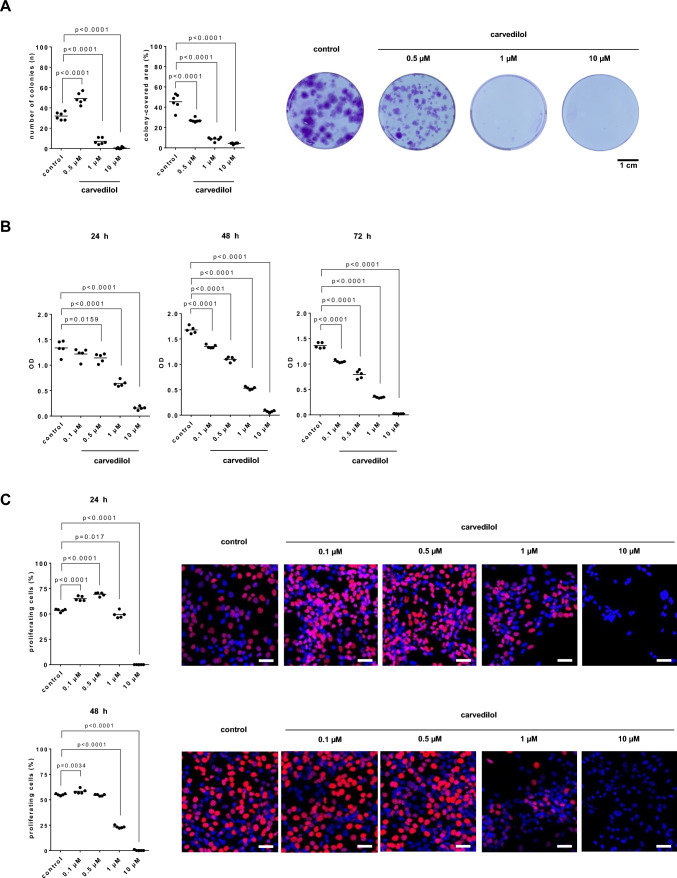
Effects of carvedilol on colony formation, viability and proliferation of WPMY-1 cells. Cell growth was determined by colony formation assays (scale bar: 1 cm), 13 d after addition of carvedilol or dimethylsulfoxide (DMSO) to controls (**A**). Viability was determined by cell counting kit-8 (CCK-8) assays, 24–72 h after the addition of carvedilol or DMSO to controls (**B**). Proliferation was determined by EdU assays, 24 h or 48 h after the addition of carvedilol or DMSO to controls (**C**). Values from totals of 5 independent experiments per series for CCK-8 and EdU, and 6 experiments for colony formation assay (one-way ANOVA with Dunnett’s test), and representative images from colony formation assays (whole wells, colonies stained darked blue) and EdU stainings (whole microscopic fields, scale bars 20 µm, nuclei of proliferating cells stained red or pink, nuclei of non-proliferating cells stained blue)

At a concentration of 500 nM, carvedilol induced bidirectional effects on colony formation of WPMY-1 cells, including increased numbers of colonies (F_3,20_ = 221.7; p < 0.0001) but reduced sizes of colonies (F_3,20_ = 126.4; p < 0.0001) (Fig. 7A, and supplementary materials Fig. 3). At concentrations of 1 µM and 10 µM, the numbers of colonies and colony-covered areas were both substantially reduced. Colony numbers amounted to 1.5 fold [1.4–1.7] of controls with 500 nM (p < 0.0001), but were reduced by 78% [68–88] with 1 µM (p < 0.0001) and by 98% [95–101] with 10 µM (p < 0.0001). Colony-covered areas were reduced by 40% [36–45] with 500 nM (p < 0.0001), 81% [77–85] with 1 µM (p < 0.0001), and 90% [92–92] with 10 µM (p < 0.0001).

### Effects of carvedilol on the viability of stromal cells

Viabilities of WPMY-1 cells were time- and concentration-dependently reduced by carvedilol, ranging from lacking and negligible effects with 100 nM and 500 nM after 24 h (F_4,20_ = 129.9; p < 0.0001), to complete reduction of viability with 10 µM after 48 (F_4,20_ = 1102; p < 0.0001) and 72 h (F_4,20_ = 703.2; p < 0.0001) (Fig. 7B). Compared to time-matched controls, decreases amounted to 9% [-2 to 19] with 100 nM (p = 0.1966), 15% [6 to 23] with 500 nM (p = 0.0159), 52% [47 to 58] with 1 µM (p < 0.0001) and 88% [85 to 91] with 10 µM (p < 0.0001) after 24 h, to 19% [17 to 22] with 100 nM (p < 0.0001), 34% [31 to 38] with 500 nM (p < 0.0001), 68% [66 to 70] with 1 µM (p < 0.0001) and 95% [94 to 97] with 10 µM (p < 0.0001) after 48 h, and to 23% [21to 25] with 100 nM (p < 0.0001), 42% [34 to 49] with 500 nM (p < 0.0001), 75% [73 to 76] with 1 µM (p < 0.0001) and 98% [98 to 99] with 10 µM (p < 0.0001) after 72 h.

### Effects of carvedilol on the proliferation of stromal cells

In the examined concentration range of 0.1–10 µM, carvedilol showed bidirectional, concentration-dependent effects on proliferation rates of WPMY-1 cells, including increases of proliferation with 0.1 and 0.5 µM and decreases with 10 µM (Fig. 7C, and supplementary materials Fig. 4). Following exposure for 24 h (F_4,20_ = 867.6; p < 0.0001), proliferation rates amounted to 1.2 fold [1.16–1.27] of controls with 100 nM (p < 0.0001), 1.3 fold [1.26–1.34] of controls with 500 nM (p < 0.0001), and 0.9 fold [0.84–1] fold of controls with 1 µM (p = 0.017), while proliferation was completely inhibited with 10 µM (p < 0.0001). The bidirectional character and extent of effects were obviously dynamic over time. Following exposure for 48 h (F_4,20_ = 1892; p < 0.0001), proliferation rates amounted to 1.1 fold [1.0–1.11] of controls with 100 nM (p = 0.0034), 0.99 fold [0.97–1.01] of controls with 500 nM (p = 0.9082), 0.4 fold [0.39–0.45] fold of controls with 1 µM (p < 0.0001), while proliferation was completely inhibited with 10 µM (p < 0.0001).

## Discussion

We observed concentration-dependent right shifts of concentration-response curves for phenylephrine, methoxamine and noradrenaline by carvedilol, with full recovery at high agonist concentrations and reflected by corresponding increases of EC_50_ values. Accordingly, carvedilol reduced neurogenic contractions by EFS, while any impact on non-adrenergic contractions was lacking. In parallel, carvedilol caused concentration-dependent effects in cultured stromal cells, pointing to bidirectional effects on growth. Antagonism of α_1_-adrenergic receptors by carvedilol has been repeatedly suggested, but rarely using smooth muscle preparations, and not from human tissues examined with physiological carvedilol concentrations. Because α_1_-adrenoceptors are a central target for medical treatment in LUTS suggestive of BPH, and as carvedilol is clinically applied as an antihypertensive and antiarrhythmic drug, which may include patients with BPH, we were interested in exploring its effects on smooth muscle contraction in the human prostate. Concentration-dependent antagonism of α_1_-adrenoceptors, even though to limited extend started with nanomolar concentrations, i.e. in the range of expectable plasma levels. Antagonism by 10 µM carvedilol was larger, with effects resembling those of clinically applied α_1_-blockers.

Carvedilol is categorized as a non-selective β-blocker, antagonizing α_1_-adrenoceptors in addition to β-adrenoceptors [9]. In radioligand competition assays, performed in HEK293 cells transfected with human adrenoceptors, K_i_ values for carvedilol amounted to 4 nM for β_1_, 10 nM for β_2_, 12.6 nM for α_1A_, 2.5 nM for α_1B_, 1.3 nM for α_1D_, and to 5 µM for α_2A_, 3.2 µM for α_2B_ and 1.3 µM for α_2C_ [20]. Similar affinities were observed in a study including β_3_, but no α-adrenoceptors, reporting K_D_ values of 1.8 nM for β_1_, 0.4 nM for β_2_, and 5 nM for β_3_ in radioligand competition assays in CHO cells transfected with human receptors [21]. The pA_2_ values for noradrenaline-induced, intracellular calcium release have been assessed in HEK293 cells expressing human α_1_-adrenoceptors, and amounted to 9.0, 10 and 10.2 for α_1A_, α_1B_ and α_1D_ [20]. In the human prostate, α_1A_ is the predominant subtype of adrenoceptors, constituting 63–85% of the total α_1_-adrenoceptors’ mRNA population in the human prostate, and accounting for smooth muscle contraction [22]. Our findings point to antagonism of α_1A_-adrenoceptors by carvedilol in human prostate tissues. Apparent pA_2_ values, calculated as an estimation for carvedilol affinity to α_1A_-adrenoceptors in our experiments ranged between 6.5 and 7.2, corresponding to estimated affinities 63–286 nM. Thus, affinities and pA_2_ values from previous biochemical and cellular assays may differ from our experiments with ex vivo tissues, with the potency in our experiments being lower but still scattering in nanomolar ranges, consistently across all analyzed series. Despite the high consistence of carvedilol effects across all three examined α_1_-agonists, 10 nM carvedilol did not affect phenylephrine- and methoxamine-induced contractions, but noradrenaline- and EFS-induced contractions. We speculate, that this difference may be attributed to divergent pharmacological profiles of α_1_-agonists. Noradrenaline activates all subtypes of adrenoceptors (though, α_1_ subtypes with highest affinity), while phenylephrine and methoxamine are synthetic, α_1_-selective ligands. Accordingly, findings were most similar between EFS and noradrenaline, and between phenylephrine and methoxamine. However, conclusions allowing detailed explanations for this different suscepetibility to 10 nM carvedilol are not possible on the basis of our findings.

The major, if not single subtype mediating prostate smooth muscle contraction is α_1A_ [2], while vasocontraction is predominantly mediated by α_1B_ or α_1D_. Three previous studies examined carvedilol effects on agonist-induced contractions of isolated vessels. Antagonism of α_1_-adrenoceptors was confirmed in rabbit aorta, where concentration-response curves for noradrenaline were right shifted to nearly one magnitude by 30 nM carvedilol and by almost two magnitudes with 1 µM [23]. In contrast, 1–10 µM carvedilol rather reduced and left shifted concentration-response curves for angiotensin II-induced contractions in canine saphenous veins, although to small extent [23]. In canine coronary arteries, 10 µM carvedilol moderately decreased U46619-induced contractions, without signs of antagonism, and substantially inhibited potassium-induced contractions [24]. In canine iliac arteries, phenylephrine-induced contractions were virtually abolished with 10 µM carvedilol, while lower concentrations were not examined [25]. Phenylephrine-induced contractions in human coronary arteries were reduced by 10 µM carvedilol in the presence of angiotensin II, while data without angiotensin II, i. e. only with phenylephrine and carvedilol were not reported from these vessels [25].

Upon activation by ligands, adrenergic receptors induce intracellular signaling by their associated, heterotrimeric G proteins imparting β-adrenergic relaxation or α_1_-adrenergic contraction. G protein-dependent receptor signaling may be interrupted by binding of β-arrestins to receptors, replacing receptor-coupled G proteins or preventing reassociation of receptors with G proteins [2]. Uncoupling and termination of G protein-mediated, receptor-induced signaling by β-arrestins represents an important mechanism of receptor desensitization [2]. In addition, β-arrestins may induce their own signaling, including β-arrestin-mediated signaling by receptors [26–28]. However, outcomes of β-arrestin-induced signaling in smooth muscle cells are not yet known. β-Arrestin-induced signaling has been severalfold related to proliferation, by various mechanisms and in different cell types, but also to mitogen-activated protein kinase signaling without functions in cell cycle [26–28]. Carvedilol is known to activate β-arrestin-induced signaling by β-adrenoceptors, following its receptor binding [12, 29, 30]. Consequently, we were interested in effects of carvedilol on growth-related functions of cultured prostate stromal cells. We observed bidirectional, and time-dependent, biphasic responses of carvedilol, including increases in colony numbers and proliferation using nanomolar concentrations, but opposing effects by higher concentrations. Opposing responses of same concentrations on growth and size of colonies may reflect opposing effects on proliferation and hypertrophy. We speculate that carvedilol responses in WPMY-1 cells included G protein- and β-arrestin-mediated components, which may differ from each other, and may depend on carvedilol concentrations and on exposure times. However, a participation of β-arrestin in carvedilol responses needs further experimentation, to be confirmed for prostate stromal cells. Finally, profound decreases in viability, seen with 1 µM and in particular with 10 µM may be provisionally explained by unspecific, cytotoxic effects, occuring independently from adrenoceptors. While decreases in viability of prostate cells may be beneficial in BPH, cytotoxic effects are in general critical. In BPH and if limited to prostate cells, both may amount to the same, i.e. to a reduction in prostate size. However, concentrations of 10 µM and systemic toxic effects can be excluded in vivo, as maximum plasma concentrations mount to 315 nM [31]. With approved standard doses, carvedilol is safe and clinically applied for treatment of cardiac failure and hypertension [31, 32], the latter being a common comorbidity of BPH.

Previous studies addressing effects on smooth muscle (or similar) cells mostly used micromolar concentrations. In hepatic stellate cells, mediating contraction in the intrahepatic vasculature, 1–5 µM carvedilol showed no or slight effects, while 20–30 µM caused profound inhibition of viability and cell cycle arrest [33, 34]. Proliferation of human pulmonary artery smooth muscle cells was unchanged, or even slightly increased by 0.1 µM carvedilol, but slightly reduced with 1 µM and nearly terminated using 10 µM [35]. Earlier studies reported inhibition of proliferation as well, using 10 µM in different lines of human vascular smooth muscle cells [36], and suggested an IC_50_ around 1 µM and reductions ≥ 65% under different conditions in rat aortic smooth muscle cells [37]. Proliferation of human pulmonary artery smooth muscle cells, induced by different growth factors was unaffected with 0.1 µM, reduced but still higher to proliferation rates without growth factors with 1 µM, and completely abolished using 10 µM carvedilol [38]. Even though most previous studies reported antiproliferative effects of carvedilol, this does not contrast our findings, as lower concentrations have not been systematically examined previously, or mostly anticipated anticontractile effects in their study designs. Besides, the effects of carvedilol may be cell type-specific. In a mouse epidermal cell line, carvedilol reduced colony formation with an IC_50_ of 243–782 nM, while viability and proliferation were reduced to a neglectable degree by 10 µM but fully by 100 µM [39]. Notably, the effect on colony formation occurred independently from β_2_-adrenoceptors, the only adrenergic receptor subtype in this cell line [39]. Together, the effects of β-adrenoceptors and carvedilol on growth-related functions are dynamic and merit further investigation, which needs to integrate the potential roles of β-arrestins in these functions.

With daily doses of 12.5 and 25 mg, plasma levels of carvedilol range from 115 to 131 nM in healthy probands, and from 256 to 315 nM in patients with chronic renal insufficiency [31]. In our experiments, right shifts of concentration-response curves and inhibition of EFS-induced contractions started with 100 nM carvedilol in all, and with 10 nM in some series. However, inhibitions resembling effects of tamsulosin or silodosin under the same conditions in our previous studies [40, 41] required 10 µM carvedilol. Thus, in the light of known plasma levels together with our findings, beginning antagonism of prostatic α_1_-adrenoceptors, and small effects on prostate smooth muscle tone may be theoretically expected with standard doses of carvedilol. With effect sizes seen with physiological carvedilol concentrations in vitro, symptom improvements in vivo may be limited, if not too small to become clinically relevant. In cardiovascular context, the clinical relevance and contributions of α_1_-adrenoceptor blockade to therapeutic effects have been questioned, and are still unclear [9, 42].

On the other hand, clinically relevant improvements in voiding symptoms by carvedilol have been reported. In hypertensive patients (n = 50) with at least moderate voiding symptoms (IPSS ≥ 12), treatment with carvedilol in hypotensive standard doses for three months reduced the IPSS on average by 4.1 points (− 32%), and increased the Q_max_ by 3.3 ml/sec (+ 25%) [43]. These changes are in full range of α_1_-blockers used for LUTS treatment [1, 2], and were not observed in a control group receiving enalapril, where improvements were limited to reduction of blood pressure [43]. A retrospective study compared the development of urological parameters, from an initial visit over a median follow-up of 36 months, in patients with BPH and palpitation receiving either carvedilol (n = 219) or another β-blocker (n = 229), and suggested benefits from carvedilol [44]. During the follow-up period, voiding scores (IPSS without questions for OAB and quality of life) increased by 5.6 points with other β-blockers, but only to 1.6 points with carvedilol, paralleled by worsening of Q_max_ with other β-blockers (− 6.1 ml/sec) but improvement of Q_max_ with carvedilol (+ 5.4 ml/sec) [44]. Another, recent crossover study including treatments with carvedilol, and terazosin combined with enalapril reported reductions of IPSS and concomitant increases in Q_max_, however, of uncertain extent [45].

In light of problems arising from polypharmacy, simultaneous improvements in blood pressure and voiding symptoms by a single compound appears attractive. The application of carvedilol for treatment of hypertension and cardiac failure requires dose titration [46]. Whether dosing of carvedilol for antihypertensive treatment by β-blockade can be balanced with doses needed for LUTS improvement by α_1_-adrenergic blockade and with side effects, by prescribers, or at all, remains elusive [46]. Tamsulosin and silodosin, the latest α_1_-blockers introduced for the treatment of voiding symptoms show the highest selectivity for α_1A_- over α_1B_- and α_1D_-receptors, which minimizes cardiovascular side effects and allows uncomplicated application for LUTS treatment [1, 2]. In turn, β-blockers are not the first-line option for drug treatment of hypertension, partly owing to their less favourable side effect profile compared to renin-angiotensin system (RAS) blockers, and to higher discontinuation rates in real-life settings, and are less widely used as RAS and calcium channel blockers [47].

We used human prostate tissues, which are evidentially characterized by high heterogeneity, due to varying degrees of BPH, different tissue compositions including varying stromal/glandular ratios, different disease conditions, and any individual heterogeneity [17, 48, 49]. Accordingly, we referred agonist- and EFS-induced contractions to contractions induced by highmolar KCl, which may reflect overall smooth muscle content and conditions of tissues, and allow to correct for heterogeneities. Samples were collected and analyzed anonymously, so that reference to age or clinical data was not possible, including preoperative treatment with α_1_-blockers, which may theoretically affect contractions in our ex vivo experiments. However, any α_1_-blocker from the preoperative medication was probably washed out during transport and storage in custodiol solution, and the equilibration, washout, and incubation periods in the organ bath. Notably, the effects seen with carvedilol were highly consistent, across different series including different α_1_-adrenergic agonists and different patients, pointing to a high validity of our findings, and to relevance across all tissue conditions in the examined patient population. In the strict sense and as another limitation, we did not specifically assess smooth muscle contractions, but contractility of prostate tissues. Thus, smooth muscle cells are the only contractile cell type in the prostate, and the predominant cell type in the stroma, but force generation of tissues may additionally depend on tissue integrity, i.e. by anchoring of smooth muscle cells in the tissue environment, including non-cellular components, glands and glandular-epithelial cells [50]. DMSO, used as solvent for carvedilol and applied to our controls may itself affect viability, and thus tissue contractions in our organ bath experiments. This was uncritical in our experiments, as tissues were still viable. Viability in the presence of DMSO was evidenced by agonist- and EFS-induced contractions (induced after application of DMSO/carvedilol), which were similar to or often even exceeded the KCl-induced contractions (assessed before application of DMSO/carvedilol). Finally, our findings from cell culture merit further experimentation and may guide further studies on apoptosis or cell death. Apoptosis or cell death may potentially account for reduced viability with 0.5 µM carvedilol, as proliferation was unchanged with this concentration.

## Conclusions

Carvedilol right shifts concentration-response curves for α_1_-agonists in human prostate smooth muscle contraction, paralleled by inhibition of neurogenic contractions. Effects start within ranges of known plasma levels, which may explain previous clinical observations, suggesting the benefits of carvedilol in voiding symptoms suggestive of BPH. In vitro, the maximum effects of carvedilol resemble those of α_1_-blockers routinely used for LUTS treatment, which requires concentrations beyond plasma levels. Carvedilol shows effects on growth-related functions of prostate cells, which are bidirectional and time-dependent, possibly reflecting G protein- and β-arrestin-mediated responses. Directed use of carvedilol for simultaneous reduction of blood pressure and voiding symptoms appears attractive but may require individual dose titration to balance cardiovascular and urological benefits, with side effects.

### Supplementary Information

Below is the link to the electronic supplementary material.

Supplementary file1 (XLSX 15 KB)

Supplementary file2 (PDF 625 KB)

## Data Availability

All data that support the findings of this study are included in this published article. Raw data are available from the corresponding author upon reasonable request.
