# Supplementary material for: Effects of carvedilol on human prostate tissue contractility and stromal cell growth pointing to potential clinical implications
Source: Pharmacol Rep. 2024 Jun 11;76(4):807–22. doi: 10.1007/s43440-024-00605-5 (PMC11294394; doi:10.1007/s43440-024-00605-5)
Supplement: Supplementary file 2 — Supplementary file2 (PDF 625 KB) [file 43440_2024_605_MOESM2_ESM.pdf]

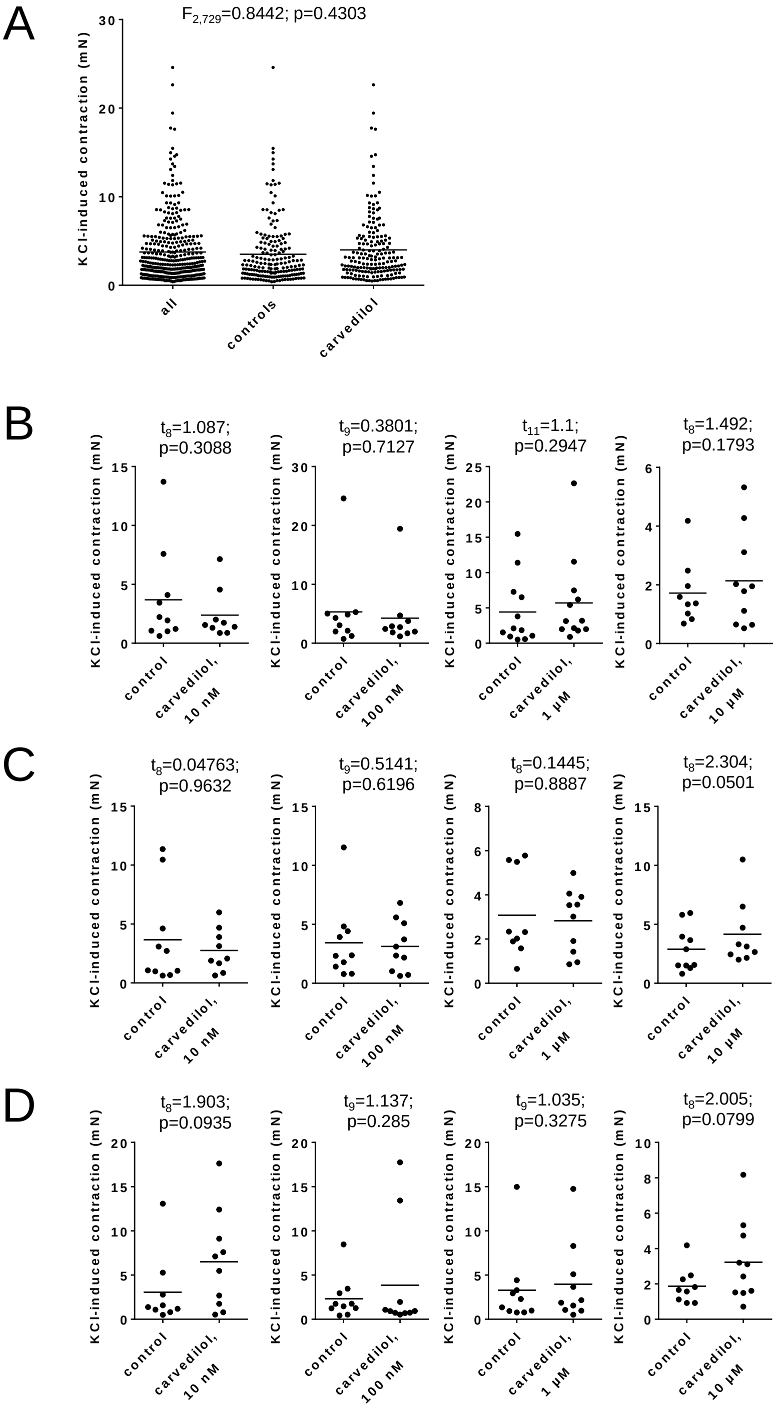

Supplementary material figure 1: KCl-induced contractions in all examined tissues (A), and in control and carvedilol groups in experiments with phenylephrine (B), methoxamine (C), and noradrenaline (D). In (A), values from all tissues are shown in the left panel, which were separated for controls and carvedilol groups in the middle and right panel. Groups were compared by one-way ANOVA with Tukey‘s test in (A), and by paired Student‘s t-test in (B) – (C).

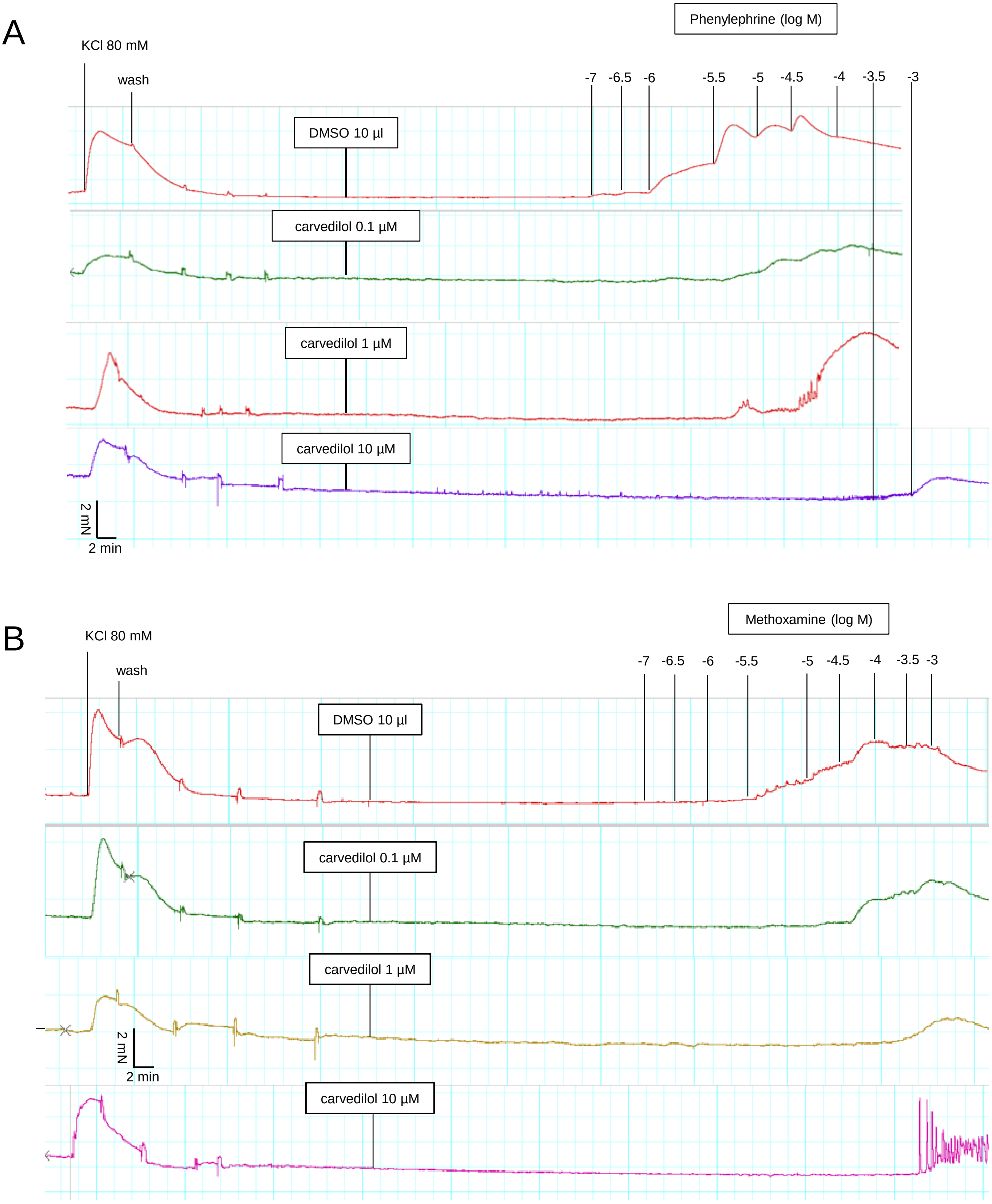

Supplementary material figure 2: Representative, original traces, from experiments with phenylephrine (A), and with methoxamine (B).

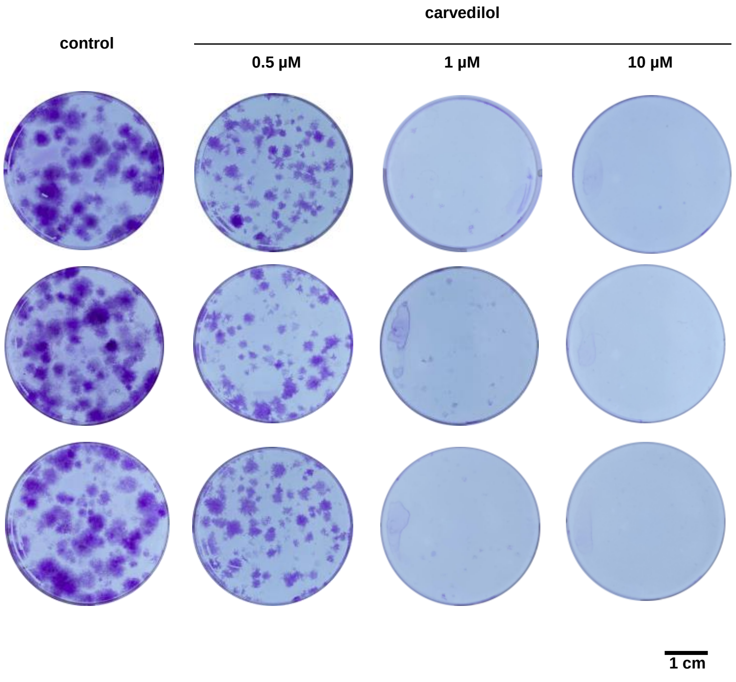

Supplementary material figure 3: Images from colony formation assays (scale bar 1 cm), after incubation with DMSO (controls), or carvedilol for 13 d. Shown are 3 images per condition, from a total of n=6 experiments, with images representing whole wells.

A

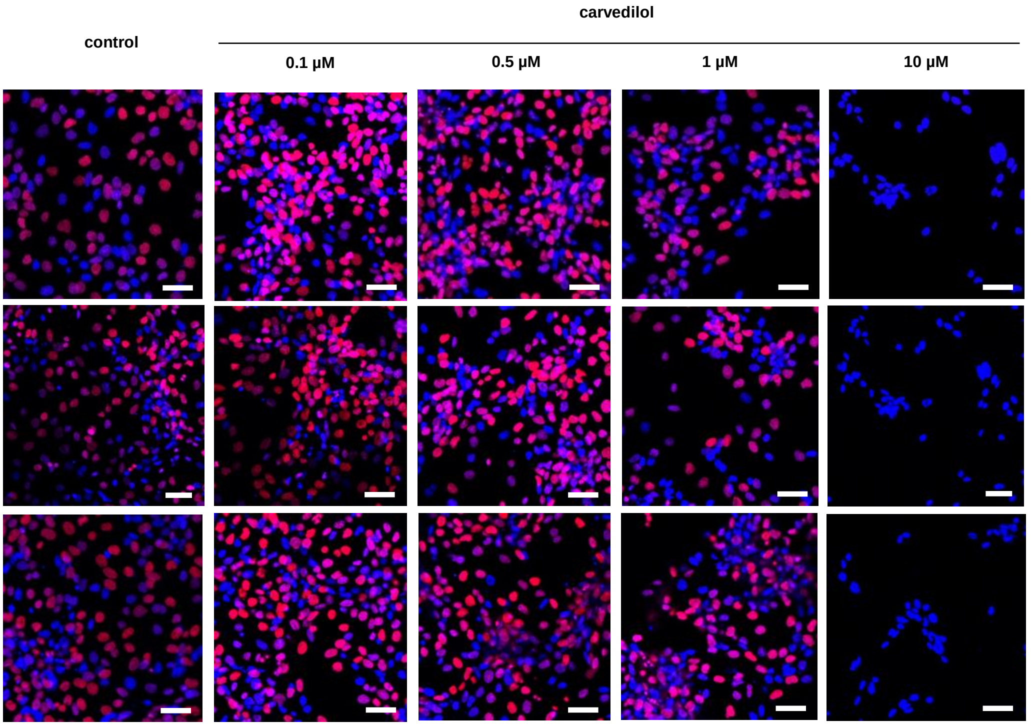

B

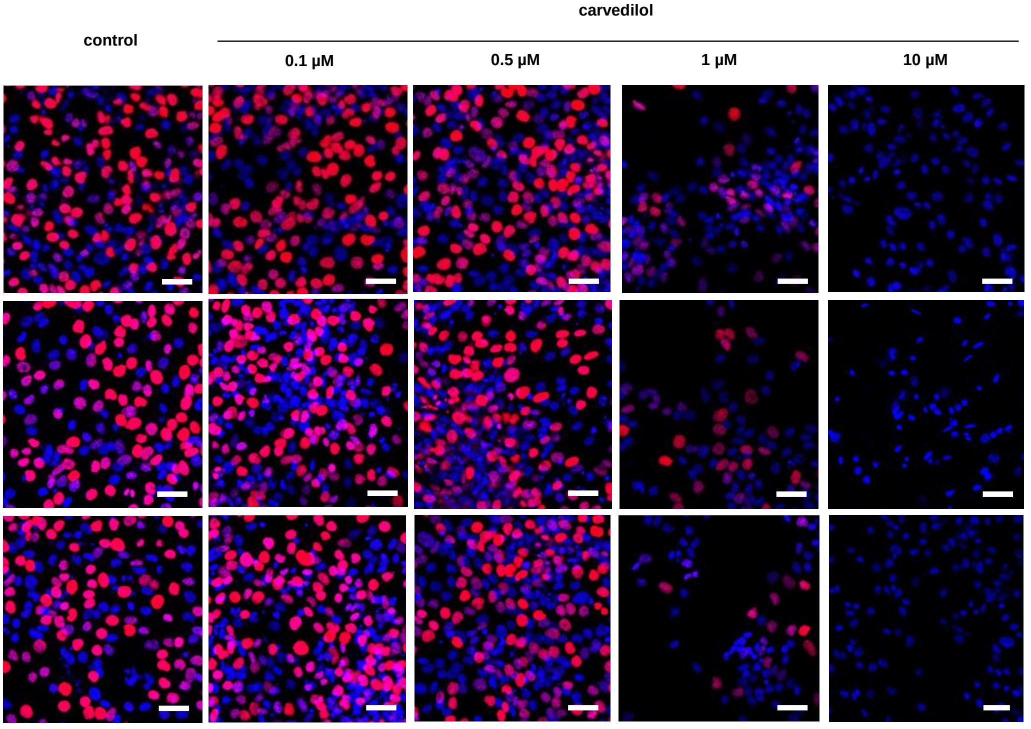

Supplementary material figure 4: Images from EdU assays (scale bar 20 µm), after incubation with DMSO (controls), or carvedilol for 24 h (A) or 48 (h). Shown are 3 images per condition, from a total of n=5 experiments in (A) and n=5 experiments in (B), with each single experiment containing a control group and all concentrations of carvedilol, and with each group determined by five-fold determination (i. e., a total of 25 pictures, from five experiments was analyzed per group).
